# Identification of biomarkers for genotyping *Aspergilli *using non-linear methods for clustering and classification

**DOI:** 10.1186/1471-2105-9-59

**Published:** 2008-01-28

**Authors:** Irene Kouskoumvekaki, Zhiyong Yang, Svava Ó Jónsdóttir, Lisbeth Olsson, Gianni Panagiotou

**Affiliations:** 1 Center for Biological Sequence Analysis, BioCentrum-DTU, Building 208, Technical University of Denmark, DK-2800 Kgs. Lyngby, Denmark; 2 Center for Microbial Biotechnology, BioCentrum-DTU, Building 223, Technical University of Denmark, DK-2800 Kgs Lyngby, Denmark

## Abstract

**Background:**

In the present investigation, we have used an exhaustive metabolite profiling approach to search for biomarkers in recombinant *Aspergillus nidulans *(mutants that produce the 6- methyl salicylic acid polyketide molecule) for application in metabolic engineering.

**Results:**

More than 450 metabolites were detected and subsequently used in the analysis. Our approach consists of two analytical steps of the metabolic profiling data, an initial non-linear unsupervised analysis with Self-Organizing Maps (SOM) to identify similarities and differences among the metabolic profiles of the studied strains, followed by a second, supervised analysis for training a classifier based on the selected biomarkers. Our analysis identified seven putative biomarkers that were able to cluster the samples according to their genotype. A Support Vector Machine was subsequently employed to construct a predictive model based on the seven biomarkers, capable of distinguishing correctly 14 out of the 16 samples of the different *A. nidulans *strains.

**Conclusion:**

Our study demonstrates that it is possible to use metabolite profiling for the classification of filamentous fungi as well as for the identification of metabolic engineering targets and draws the attention towards the development of a common database for storage of metabolomics data.

## Background

Functional genomics approaches are increasingly being used for the elucidation of complex biological questions with applications that range from human health to microbial strain improvement [1-3]. Functional genomics tools have in common that they aim to map the complete phenotypic response of an organism to the environmental conditions of interest. Metabolomics technology is used to identify and quantify the metabolome, which represents the dynamic set of all small molecules – excluding those resulting from DNA and RNA transcription or translation – present in an organism or a biological sample [4]. Fundamentally, the measured metabolite levels at a defined time under specific culture conditions for a given genotype should reflect a precise and unique signature of the metabolic phenotype [5]. In this sense, the technique is distinct from metabolic profiling, which looks for target compounds identified *a priori *and their consequent biochemical transformation. Metabolomics has proven to be very rapid and superior to any other post-genomics technology for pattern-recognition analyses of biological samples. One of the major advantages of metabolomics is that there are fewer metabolites than genes or proteins, resulting in significant data reduction and high-throughput analysis. Furthermore, some environmental perturbations or genetic manipulations do not result in significant alterations at transcriptome and/or proteome levels; however, significant detectable changes in metabolite concentrations may be observed [6]. Quantitative assessment of metabolite concentrations enables decoupling from genetic or environmental perturbations that may not affect gene transcription and/or protein translation, but may for example affect enzyme activity levels that could lead to correspondingly more or less metabolite. Metabolomics is therefore considered to be in many senses, more discriminatory than transcriptomics and proteomics.

The application of biostatistics and novel data-handling frameworks will have a strong role in the extraction of biologically meaningful information from large metabolomic data sets. Traditionally, data analysis has been conducted using methods that look for linear relationships within the metabolomics data, like principal components analysis (PCA) [7-9]. In recent years, non-linear methods have been successfully applied on analysis of metabolomics data, including clustering methods, e.g self organizing maps (SOM) [10], as well as classification methods, e.g back propagation artificial neural networks [11] and decision trees [12]. The results from these analyses look promising and indicate that there indeed are non-linear patterns within the data. Like PCA, SOM is a tool for visualizing data sets and for extracting high-value features using unsupervised approaches, which are helpful to experimentalists for subsequent data interpretation. Clustering or unsupervised data analysis relies on similarities in unlabeled data, -in this case the metabolite concentrations and not on a preset class or target value as in classification or supervised data analysis. Given that there is no initial bias based on required model assumptions like in supervised methods, unsupervised methods are far less likely to identify false correlations. If an unsupervised algorithm clusters independent metabolome data with a high or low degree of separation, then the confidence associated with reporting identifying highly-correlated or un-correlated biological data, respectively, is high.

One of the more highly valued features of filamentous fungi is their capacity for producing a great variety of secondary metabolites. Several of these compounds are currently produced commercially, such as various antibiotics, vitamins, and value-added chemicals. For example, Aspergilli serve as microbial cell factories that have been metabolically engineered for the production of organic acids [13], enzymes [14] and polyketides, such as statins – amongst the highest-value pharmaceutical class of compounds primarily produced by A*spergillus terreus *[15]. Included in this genus is *Aspergillus nidulans *representing an important model organism for studies of cell biology and gene regulation. In the present investigation we have exploited a metabolomics approach to search for high-value phenotypic features, we refer to as biomarkers, in recombinant *Aspergillus nidulans*. The strains investigated are *A. nidulans *mutants, resulting from metabolic engineering efforts to produce the 6- methyl salicylic acid polyketide molecule. Metabolic engineering seeks to identify, introduce, and enhance those gene products that are important in increasing the productivity of biological processes, and to manipulate their concentrations or activities accordingly [16]. Our approach consists of two analytical steps, an initial non-linear unsupervised analysis (SOM) to cluster the metabolome data collected from well-defined cultivations of the investigated strains, followed by a second, supervised analysis for training a predictor built on selected biomarkers. Identification of biomarkers, where high-value information is concentrated and stored, will subsequently suggest that the bulk of regulatory nodes are centered on these metabolites. Regulation, defined in this context as the metabolic response to a stimulus, is a primarily differentiator of organisms. Metabolic engineering aims to identify, isolate, and augment those regulatory points to enhance production of a desired product.

## Results

### Preprocessing of data

The initial preprocessing for data reduction revealed seven metabolites as being most significant for discriminating the four *A. nidulans *strains, and three metabolites for discriminating among the four carbon sources (glucose, xylose, glycerol and ethanol), as shown in Table 1. From the above metabolite set only four of the ten compounds could be identified using the in-house metabolite library (valine, 6-MSA, lactic acid, fumaric acid). These sets of metabolites were obtained by applying the combination of *CfsSubsetEval *and *BestFirst. CfsSubsetEval *prefers sets of descriptors that are highly correlated within a class, referred to as intra-correlation, but have relatively low inter-correlation. *CfsSubsetEval *was combined with the *BestFirst *search function that performs greedy hill climbing with backtracking. *BestFirst *is a heuristic algorithm that makes at each stage the local optimum choice with the hope of finding the global optimum. It starts with the full set and deletes descriptors one at a time (backtracking, or backward elimination).

**Table 1:** a) The seven biomarkers in respect to discrimination of the four *A. nidulans *strains, b)The three biomarkers in respect to discrimination of the four cultivation conditions (glucose, xylose, glycerol and ethanol as carbon sources)

Metabolite (a)	Metabolite (b)
*M19: unidentified*	*M4: lactic acid*
*M20: valine*	*M118: unidentified*
*M23: unidentified*	*M157: fumaric acid*
*M84: unidentified*	
*M92: unidentified*	
*M238: unidentified*	
*M350: 6-methyl salicylic acid (6-MSA)*	

The other combinations that were evaluated, gave either the same set of metabolites as before or larger sets (sets of 36 and 11 metabolites, for strain and carbon source discrimination respectively) that included all the metabolites shown in Table 1. As it is preferable to work with as few biomarkers as possible, the smaller sets were chosen for the further modeling steps.

### Clustering

**Figure 1:**
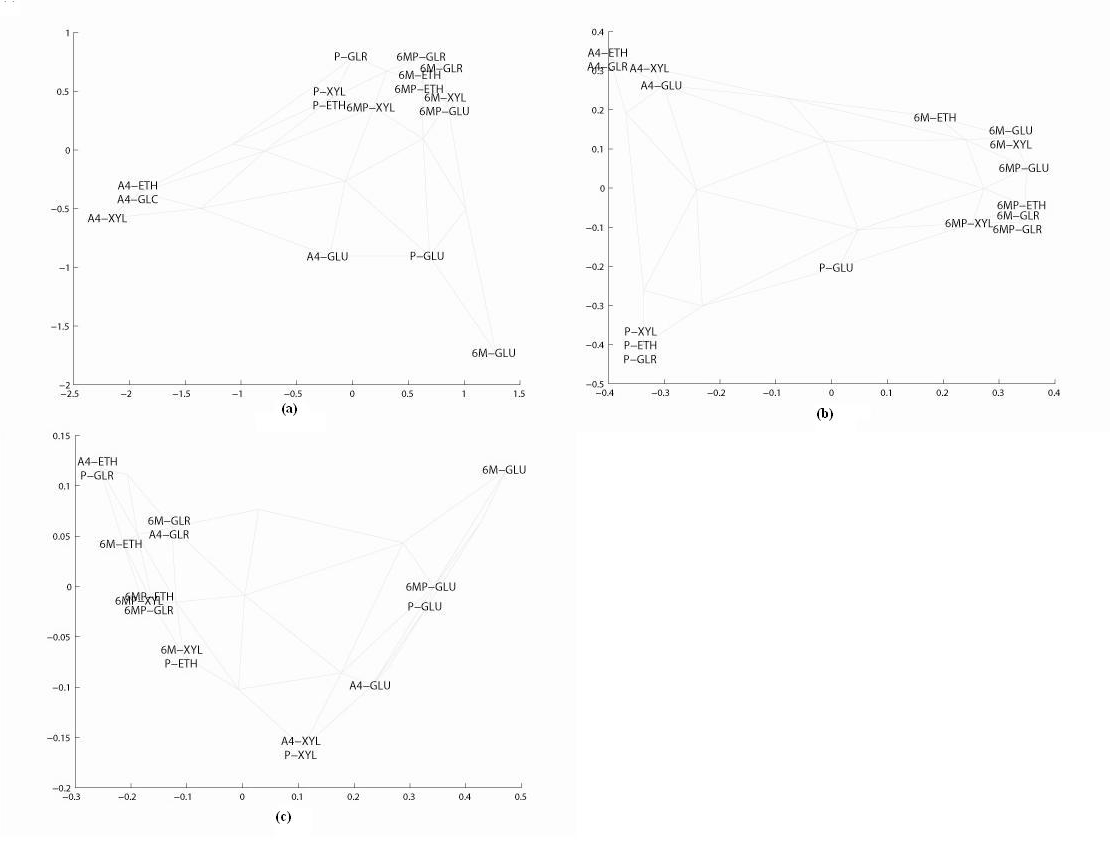
(a) SOM clustering based on all 464 metabolites detected after cultivation of wild type and recombinant *A. nidulans *strains on glucose (GLU), xylose (XYL), glycerol (GLR) and ethanol (ETH). (b) SOM clustering based on the seven metabolites from Table 1a. (c) SOM clustering based on the three metabolites from Table 1b. A4: *A. nidulans *A4 strain, P: AR1*phk*GP74 strain, 6M: AR1*6msa*GP74 strain, 6MP: AR1*phk6msa*GP74 strain. Grey lines denote the topological relations between the neurons on the SOM grid.

In contrast to supervised methods that weigh the single descriptor based on relevance, SOM treats each descriptor equally. Therefore, a combination of well and poorly performing descriptor vectors is not recommended when applying SOM [17]. The importance of data reduction is demonstrated in Figure 1, where clustering is performed based first on the whole set of detected metabolites (Fig. 1a), and subsequently using the seven and the three selected metabolites (Fig. 1b and 1c). Figures 1a–c show mapping of the high dimensional data from SOM in a two dimensional space, using a PCA-like projection of the descriptor vectors, where distances between the samples can be more easily visualized.

When all the metabolites are used, discrimination of the samples is not possible either based on genotype or by cultivation condition (Fig. 1a). When the seven selected metabolites are employed, clustering based on the different genotypes provides a high degree of correlated discrimination (Fig. 1b). In Figure 1b, the samples of the *A. nidulans *A4 strain are clustered together, and so do the samples of the AR1*phk*GP74 strain. It is worth noting that in both cases, the strains cultivated on glucose are furthest from their cluster centers and approach each other. Although the glucose to glucose inter-cluster distance is longer than the intra-cluster distance, the data suggests a stronger correlation across the two different strains cultivated on glucose compared to the other three carbon sources. AR1*6msa*GP74 and AR1*phk6msa*GP74 strains form a distinct cluster, distant from the other two, with very short inter-cluster distances suggesting strong similarity of the two strains.

When discrimination of the samples based on the carbon source (using the three selected metabolites of Table 1b) is attempted (Fig. 1c), the SOM grid seems distorted and the clustering is relatively poor. All strains cultivated on glucose and two strains (*A. nidulans *A4 and AR1*phk*GP74) cultivated on xylose are forming distinct clusters whereas there is no discrimination in the metabolic signature of cells grown on ethanol or glycerol. This suggests that the genotype is a much stronger distinguishing feature than the carbon source used for cultivation of the different *A. nidulans *strains when metabolite profiles are considered.

**Figure 2:**
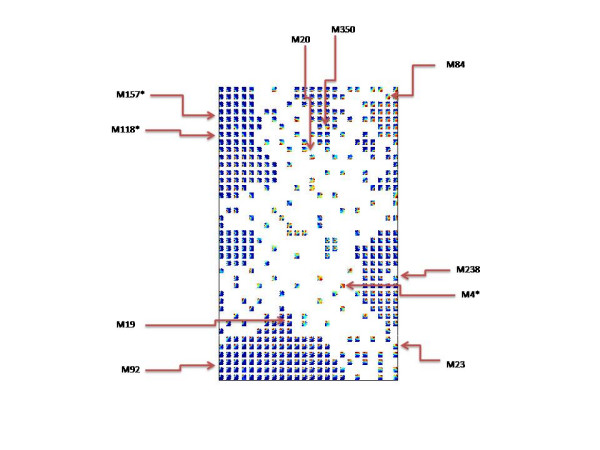
Clustering of all 464 component planes (metabolites) based on similarity in the distribution profiles of the vector values of the respective metabolites over the data set. Color key: blue- low values, red- high values. M19, M20, M23, M89, M92, M238, M350 are the seven biomarkers in respect to discrimination of the four *A. nidulans *strains. M4*, M118*, M157* are the three biomarkers in respect to discrimination of the four cultivation conditions (carbon sources).

Figure 2 visualizes the component plane matrix, where each plane shows the range of values of one metabolite in the clustered data set (color range from blue to red corresponds to a value range from low to high, respectively). The metabolites of immediate interest are those with values demonstrating high degrees of sensitivity to the genotype, which we speculate exert the control over regulatory biological networks. Therefore, those metabolites represented by phase planes with the highest spectrum of color range are indicative of metabolites with the highest degree of variance and suggest highly concentrated nodes of biological information.

Furthermore, in Figure 2 the component planes are clustered based on similarity in the distribution profiles of the component vectors over the data set, which allows us to draw interesting conclusions regarding the output of the data reduction step described previously. As seen in the figure, there are seven distinct clusters that include the majority of the 464 metabolites being placed on the borders of the matrix. Each cluster contains metabolites that are highly correlated with each other. An interesting observation is that all the seven metabolites of Table 1a belong to six clearly distinguished large clusters of highly correlated metabolites, with profiles that show quite high variance.

On the other hand, two of the three metabolites of Table 1b come from the same cluster of low variance metabolites (top left), while the third one has a totally unique profile and is therefore placed on its own in the central part of the matrix. This explains the inability of these three metabolites to cluster the data based on the different carbon source used in the cultivation (Fig. 1c).

**Figure 3:**
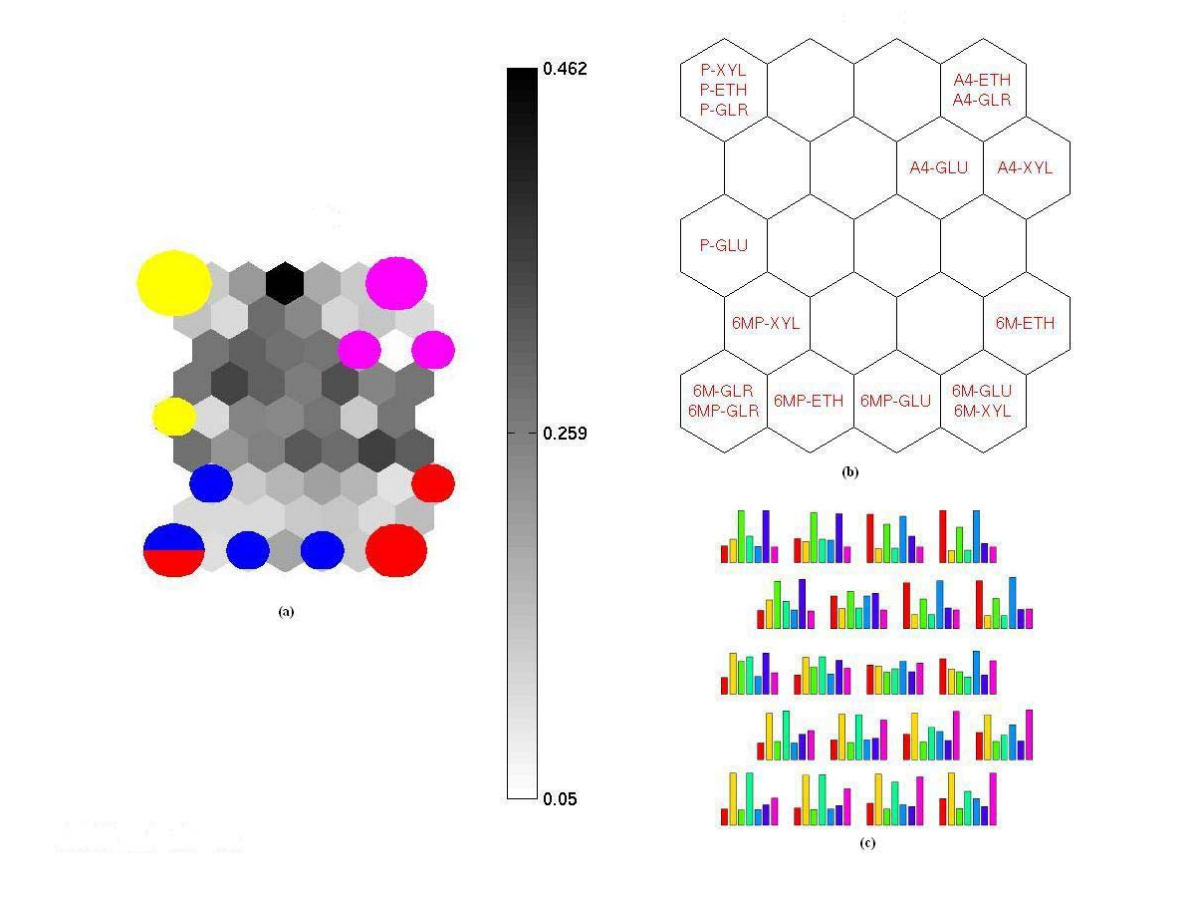
(a) U-matrix showing the distance between the neurons (b) Labels map showing the clustering of the samples based on the seven metabolites from Table 1a. glucose (GLU), xylose (XYL), glycerol (GLR) and ethanol (ETH). Color key: magenta- *A. nidulans *A4 strain (referred in the figure as A4), yellow- AR1*phk*GP74 strain (referred in the figure as P), red- AR1*6msa*GP74 strain (referred in the figure as 6M), blue- AR1*phk6msa*GP74 strain (referred in the figure as 6MP). (c) Map prototype vectors are bar charts showing the distribution of the values of the seven metabolites on the untrained map, the coordinates of the map on seven dimensions. Color key: red- M19, yellow- M20, green- M23, light blue- M89, blue- M92, purple- M238, magenta- M350.

In order to analyze further the clustering based on the seven selected metabolites, Figures 3a–c were created. The unified distance matrix (U-matrix) of Figure 3a makes a 2-dimensional visualization of the distance between the neurons, where different shades of grey are used to separate the neurons that are "near" to one another (white-light grey) to neurons that are "far" or "distant" from one another (dark grey-black).

The label map of Figure 3b makes a 2-dimensional visualization of the information from all the component planes and shows the clustering of the samples based on the seven selected metabolites (Table 1a). It should be noted that information in Figures 3a–b is equivalent to Figure 1b, with the distance between neurons visualized by grey-scale in one case (Figures 3a–b) and by lines in the 2D-space in the other (Figure 1b).

Looking at the U-matrix and labels map of Figure 3, it is worth noting that in the case of AR1*6msa*GP74 and AR1*phk6msa*GP74 strains, the cultivation condition is a stronger discriminative parameter than the type of strain (6M-GLR and 6MP-GLR are placed in the same neuron, while 6M-GLU and 6MP-GLU are at neighboring neurons in a light-gray area of the map).

The bar-planes of Figure 3c visualize the map prototype vectors (i.e. the coordinates of the map) as bar charts, indicating which metabolic signatures/profiles are responsible for clustering samples in each neuron. According to the bar-planes, the high concentrations of M23 and M238 are responsible for the clustering of the three samples of the AR1*phk*GP74 strain at the top left corner of the labels map. Similarly, the high concentrations of M19, M23 and M92 are responsible for the clustering of the samples of the *A. nidulans *A4 strain at the top right corner of the labels map.

### Classification

The observation of the natural clustering of the samples is a guide towards whether it is feasible to model the genotype or the used carbon source based on alterations in the metabolite profile. From the above analysis it appears that an accurate predictor of the samples' cultivation condition cannot be built based on the given information. The analysis reveals that the different strains do form quite distinct natural clusters, suggesting that the metabolites that characterize each sample may be used as model parameters for the prediction of the genotype.

Table 2 lists the performance of the four models on classifying the 16 given samples according to genotype, based on the seven selected metabolites. A first conclusion is that linear models (Linear Perceptron and Logistic) perform worse than the non-linear (Multilayer Perceptron and SMO). This indicates that the given classification task calls upon non-linear relationships and integration of data, often not present in simpler models. More specifically, the Logistic model classifies only half of the samples correctly. The Multilayer Perceptron with zero hidden layers manages to correctly classify 11 out of the 16 samples. When we add one hidden layer to the neural network, the model becomes non-liner and its performance is slightly improved. However, the best performance comes from the Support Vector Classifier, which correctly classifies 14 out of the 16 samples.

**Table 2:** Comparison of performance of linear and non-linear machine-learning methods

	Correctly classified samples
Support Vector Classifier	14/16 (87.5%)
Multilayer Perceptron (1hidden layer with 4 neurons)	12/16 (75.0%)
Multilayer Perceptron (no hidden layers)	11/16 (68.8%)
Logistic	8/16 (50.0%)

Looking into the output of the Support Vector Classifier in more detail (confusion matrix of Table 3), it manages to correctly classify all samples that belong to the *A. nidulans *A4 and AR1*phk*GP74 strains, but misclassifies one AR1*6msa*GP74 sample as AR1*phk6msa*GP74 and one AR1*phk6msa*GP74 sample as AR1*6msa*GP74. This is not surprising, considering the similarity of the two strains that was observed in the previous clustering routine.

**Table 3:** Confusion matrix of Support Vector Classifier for the four *A. nidulans *strains

AR1*6msa*GP74	AR1*phk6msa*GP74	*A. nidulans *A4	AR1*phk*GP74	←classified as
3	1	0	0	AR1*6msa*GP74
1	3	0	0	AR1*phk6msa*GP74
0	0	4	0	*A. nidulans *A4
0	0	0	4	AR1*phk*GP74

### Biological Significance

One of the primary objectives of metabolomics is to contribute to the design and implementation of metabolic engineering strategies in potential industrial hosts. There is often a disconnection between large-scale omics data sets and interpretation of the data in a physiological context that permits rational genetic or biochemical engineering applications. Tables 1a and 1b provide a summary of the seven and three biomarkers detected for discrimination of the four *A. nidulans *and four carbon substrates, respectively. It is interesting to note that of the seven biomarkers listed in Table 1a, two could be identified based on information in our in house library being valine (M20) and 6-MSA (M350). It is intuitive, yet none the less significant, that 6-MSA was identified as a biomarker metabolite across the four strains, confirming the detectable relationship between intentional genetic manipulations and resulting metabolite profiles. However, the other identified metabolite, valine, also provides some interesting insight into discrimination of the four strains. Valine, a branched, non-polar, amino acid, is coupled to the isoleucine and leucine super-family synthesis pathways. The first reaction in valine synthesis is a decarboxylation of pyruvate to form acetolactate, catalyzed by acetolactate synthase (E.C. 2.2.1.6). One of valine roles is as the primary substrate in the biosynthesis of Co-enzyme A. In Table 1b, two metabolites are identified as discriminators of the four culture conditions: lactic acid (M4) and fumaric acid (M157). It's interesting to note that both metabolites, similar to valine, utilize pyruvate as their primary substrates. Lactic acid is formed by the NADH catalyzed reduction of pyruvate by lactate dehydrogenase (E.C. 1.1.1.28), while fumaric acid is formed by the oxidation of succinate, coupled to the reduction of FADH_2_, by succinate dehydrogenase (E.C. 1.3.99.1), as an integral part of the Krebs cycle. Pyruvate enters the Krebs cycle utilizing acetyl-CoA as an essential co-factor. It is further interesting to note that 6-MSA utilizes acetyl-CoA as an essential co-factor in its biosynthesis. It is expected that the four carbon sources utilized, coupled with the four mutant strains evaluated, would significantly impact pyruvate metabolism, which serves as key regulatory node for balancing purely fermentative and respiro-fermentative metabolism. However, identification of valine, lactic acid, and fumaric acid as key biomarkers provides highly specified targets for further investigation and development of potential metabolic engineering strategies. For example, increasing 6-MSA production would be the likely require the flux through valine biosynthetic pathways to increase to boost acetyl-CoA pools, while decreasing the flux from pyruvate to lactate, would likely result in increased flux through the Krebs cycle, forming the required intermediates, such as 2-oxoketoglutarate and glutamate, for valine biosynthesis. Searching for information rich metabolic nodes derived from a combinatorial survey of different culture conditions and genotypic organisms provides information and non-intuitive targets not decipherable from a simple inspection of known biochemical pathways.

## Discussion

In this study, we investigated metabolomic profiles of different *A. nidulans *strains, wild-type and mutants grown on a diverse array of carbon sources. This investigation reports a successful approach for developing a biomarker metabolite set that captures much of the metabolite variation, and consequently, high-value, discriminatory information present in the different *Aspergilli sp*. metabolome profiles using SOM and SMV. The principal objective of SOM is to obtain a 2D projection of a multidimensional space. This projection keeps the topology of the multidimensional space, i.e., points which are close to one another in the multidimensional space are neighbors in the two-dimensional space as well. The training of the network is unsupervised, that is, the property of interest, in this case the genotype, is not used during the training process. In the course of training, the objects are randomly presented to the neural network in an iterative manner. For each iteration step the so-called winning neuron for the input object is identified by determining the neuron having the minimum Euclidean distance to the input objects, i.e. the concentration profile of metabolites in each sample. To improve the response of the network, the neuron weights are adapted to become more similar to the input pattern. After termination of training, the response of the network is calculated for each object in the data set. The projection of the data set into the 2D space is then performed by mapping each object into the coordinates of the winning neuron [18]. The SOM has already been widely applied in engineering [19] and many other fields [20] and is gaining popularity in the fields of medicine, computer-aided diagnosis and biotechnology [[21-23], respectively]. In our study, SOM was proven an invaluable tool to reveal a holistic picture of metabolism and provide insight into the relationships between the concentration levels of a metabolite pool and the genotype. In Figure 1b, there is a clear cluster of the *A. nidulans *A4 wild type as well as the AR1*phk*GP74 strain, however, when the strains were cultivated on glucose they are displaced furthest from their cluster centers, and closer to one another. This is not surprising since the physiological characterization of the AR1*phk*GP74 mutant has shown that overexpression of the phosphoketolase gene has significant effects on the specific growth rate on xylose, glycerol and ethanol but no effect on glucose [24]. On the other hand, it is obvious that the insertion of the gene coding for the secondary metabolite 6-MSA (strains AR16msaGP74 and AR16phk16msaGP74) resulted in mutants with very distinct metabolite profiles (Figure 1b). The concentrations of metabolites in the central carbon metabolism are relatively constant, while the concentrations of metabolites that are present in pathways of secondary metabolism demonstrate much larger concentration ranges. The dominant role of secondary pathways for metabolite discrimination between genotypes was further verified by the selection of 6-MSA as a biomarker (Table 1a). The inability of SOM to differentiate the metabolite profile of the two mutants AR1*6msa*GP74 and AR1*phk6msa*GP74 grown on glycerol is in agreement with our findings from the physiological characterization where the production of 6-MSA of cells grown on this carbon source was very low [24]. Metabolic flux analysis of the AR1*6msa*GP74 mutant has shown that the insertion of the 6-MSA gene increased the flux through the phosphoketolase pathway due to increased requirements for the acetyl-CoA precursor molecule [24]. This supports our findings from the metabolite profile study that the two mutants AR1*6msa*GP74 and AR1*phk6msa*GP74 have a very similar metabolic signature (Fig. 1b).

A very interesting result was that the biomarker selection by the neural network was not only based on the discrimination power but also on the interconnection with other metabolites that show similar variation (Fig. 2). Selection of biomarkers that belong to larger metabolic networks tightly connected could be invaluable for the identification of regulatory nodes- a core element of metabolic engineering.

SMV is a supervised learning method that performs nonlinear mapping of input data that are inseparable in a low dimensional space, to a higher dimensional space, where a maximal separating hyperplane is constructed. As 'support vectors' are considered the samples along the hyperplanes that are used to generate the maximum margin hyperplane between the two classes. Selecting this particular hyperplane maximizes the SMV's ability to predict the correct classification of previously unseen data. This technique differentiates SMV from other hyperplane based classifiers and seems to be its key to success. An excellent and detailed description of how support vector machines work can be found in [25]. SVM in our study was employed to construct a predictive model capable of distinguishing between different *A. nidulans *strains based on their metabolome profile. We were able to validate significant differences in metabolite levels and to detect metabolic signatures that classify correctly 90% of the strains. However, what still remains a challenge is to "decode" the selected biomarker set since six from the ten compounds could not be identified using our "in house library" (consisting of 78 metabolites), showing how important it is to develop a common database to store metabolomics data.

## Conclusion

In this work, is to our knowledge the first time that a broad metabolite profile analysis was applied to *A. nidulans *or any other *Aspergilli sp*., which, when combined with mathematical models and statistical assessment, allowed us to reach a higher level of biological understanding. Metabolic fingerprinting and biomarker identification have numerous established pharmaceutical applications, but are only recently starting to be exploited for development and enhancement of metabolic engineering strategies applied to industrial microorganisms. Identification of a limited number of metabolites where high-value information is stored essentially suggests that the bulk of regulatory nodes are centered around these metabolites. Regulation, specifically the metabolic response of an organism to a stimuli (genetic or environmental), is a discriminatory feature of microorganisms. Therefore, metabolic engineering aims to identify those regulatory points and manipulate them to enhance production of a desired product. With a biomarker set available one could immediately identify all metabolic pathways leading to the formation and consumption of that metabolite to:

○ focus high-level gene annotation, ensuring that those pathways are well defined;

○ include them in genome-scale models for simulation purposes to determine if, via stochiometry, the final product formation can be enhanced;

○ over-express or delete using a factorial design to determine if within the biomarker set which metabolite exerts the most metabolic control; and,

○ introduce non-native pathways from other organisms to further push the limits of production.

Furthermore our study demonstrates that it is possible to use metabolite profiling for the identification and classification of filamentous fungi.

## Methods

### Strains

Four strains were used in the present study; the *A. nidulans *A4 wild type, the *A. nidulans *AR1*phk*GP74, where the gene (XP_662517) encoding phosphoketolase has been overexpressed, as well as the two mutants AR1*6msa*GP74 and AR1*phk6msa*GP74 (double mutant) that contain the P22367 gene encoding for the 6-MSA polyketide molecule. The construction of the strains has been described elsewhere [24].

### Growth and culture conditions in fermentors

For all the *A. nidulans *cultivations a chemically defined medium containing trace metal elements was used. The medium used had the following composition: 15 g (NH_4_)_2_SO_4 _l^-1^, 3 g KH_2_PO_4 _l^-1^, 2 g MgSO_4_.7H_2_O l^-1^, 2 g NaCl l^-1^, 0.2 g CaCl_2 _l^-1 ^and 1 ml trace element solution l^-1^. Trace element solution composition (per litre): 14.3 g ZnSO_4_.7H_2_O, 13.8 g FeSO_4_.7H_2_O and 2.5 g CuSO_4_.5H_2_O. Arginine, 0.7 g/L, was added in the auxotrophic strains (AR1*phk*GP74 and AR1*6msa*GP74) by sterile filtration. The carbon sources used were glucose, xylose, glycerol and ethanol (20 g l^-1^) respectively. To determine the metabolite profiles cultivations were performed in well-controlled 1.5 l bioreactors with a working volume of 1.2 l. The bioreactors were equipped with two disc-turbine impellers rotating at 350 r.p.m. The pH was controlled at 5.5 ± 0.1 by addition of 2 M NaOH or HCl, and the temperature was controlled at 30 ± 0.1°C. Air was sparged through a ring-sparger for aeration of the bioreactor at a constant flow rate of 1.0 vvm (volume of gas per volume of liquid per minute).

### Cell mass determination

Cell dry weight was determined using nitrocellulose filters (pore size 0.45 μm, Gelman Sciences). The filters were pre-dried in a microwave oven at 150 W for 15 min and subsequently weighed. A measured volume of cell culture was filtered and the residue was washed with distilled water and dried on the filter for 15 min in a microwave oven at 150 W. The filter was weighed again and the cell mass concentration was calculated.

### Sampling, extraction and determination of intracellular intermediary metabolites

For the analysis of intracellular metabolites triplicate samples were collected at the middle of the exponential growth phase. 10 ml fermentation broth was immediately quenched in 20 ml of cold 72% methanol (-40°C). After quenching the cells were separated from the quenching solution by centrifugation at 10000g for 20 min at -20°C and the intracellular metabolites were extracted as described by Villa-Boas et al. [26]. Finally the samples were lyophilized and stored at -80°C until further analysis. The lyophilized samples were derivatized using methyl chloroformate as described by Villas-Boas et al. [27]. Amino and non-amino organic acids were analysed by GC-MS. GC-MS analysis was performed with a Hewlett-Packard system HP 6890 gas chromatograph coupled to a HP 5973 quadrupole mass selective detector (EI) operated at 70eV. The column used for all analyses was a J&W1701 (Folsom, CA, 30-m × 250-μm-0.15 μm film thickness). The temperature of the inlet was 180°C, the interface temperature was 230°C, and the quadrupole temperature was 150°C. The profile of identified intracellular amino and non-amino organic acids was expressed in peak areas normalized by the biomass (Additional file 1).

### Computational methods

The data from GC-MS analyses were deconvoluted using the AMDIS spectral deconvolution software package [28]. SpectConnect was used to automatically catalog and track otherwise unidentifiable conserved metabolite peaks across sample replicates and different sample conditions groups without use of reference spectra [29]. Using SpectConnect 464 metabolite peaks (referred to from now on as M1-464) were detected and more than 40 were identified using an in-house library. Clustering and classification tools were used for the identification of specific differences between metabolite profiles and the characterization of specific biological activities. In the analysis each sample corresponds to a different genotype (*A. nidulans *A4, AR1*phk*GP74, AR1*6msa*GP74, and AR1*phk6msa*GP74) each cultivated on previously specified carbon source (i.e., glucose, xylose, glycerol, ethanol, respectively).

### Preprocessing of data

Due to the large number of available descriptors (concentrations of the different metabolites) compared to the data set (number of mutants cultivated in different carbon sources), data reduction was considered necessary in order to remove irrelevant and/or intercorrelated descriptors and noise. For this purpose, model training was preceded with a descriptor selection stage in order to eliminate all but the most relevant descriptors. Reducing the dimensionality of the data by removing unsuitable descriptors usually improves the performance and speed of learning algorithms, and most importantly, yields a more compact and easily interpretable representation of the relationship between the input and output data.

In this work, data reduction was done using the freely available Java software package WEKA (version 3-4-6) [30]. The data reduction was done with the *CfsSubsetEval *descriptor subset evaluator, in combination with two different search algorithms, *BestFirst *and *GreedyStepwise*. These algorithms use greedy hill climbing with and without backtracking, respectively. *CfsSubsetEval *was chosen due to its ability to estimate the predictive value of all the descriptors individually and, at the same time, to evaluate the degree of redundancy among them. Data reduction was also attempted with three different single-descriptor evaluators, namely *ChiSquaredAttributeEval, Symmetrical *and *InfoGain *combined with the *Ranker *ranking method.

### Clustering

*Self-Organizing Maps *(SOM) [31] were applied for the clustering of the metabolome data using the Matlab SOM-Toolbox [32]. The SOM Toolbox is a function library for the Matlab 5 computing environment, required for implementing the SOM algorithm and its visualization. It is currently in version 2.0 beta and is publicly available at [33].

The normalization of the input data and the initialization of training were optimized based on the obtained quantization error after training. The logistic transformation (scaling of all values between [0 1]) and linear initialization of training produced the lowest quantization error. For the training of SOM the default parameters were used: hexangular map lattice with unconnected edges, batch training mode, and inverse function learning rate. A map size of 5 × 4 was chosen automatically by SOM based on the dimensions of the input data. The training length was set to 20 epochs (iterations), based on the point that the calculated quantization error stabilized.

### Classification

Two linear and two non-linear classifiers were selected from the WEKA toolbox to be trained for the classification of the data set; Logistic, Multilayer Perceptron (in both its linear and non linear form) and SMO [34].

*Logistic*, is a linear, multinomial logistic regression model. *Multilayer Perceptron *is a back-propagation neural network. However, the network is readily transformed to linear when trained with zero hidden neurons. The optimized parameters for the non-linear Multilayer Perceptron are shown in Table 4. The learning rate corresponds to the amount the weights of the hidden neurons that are being updated, and the momentum is the weight applied during updating.

**Table 4:** Optimized parameters for Multilayer Perceptron

*Training time:*	100 epochs (iterations)
*Learning rate:*	0.3
*Momentum:*	0.2
*Hidden layers/neurons:*	1/4
*Validation method:*	Leave-one-out cross-validation

*SMO *is a non-linear method that implements the *S*equential *M*inimal *O*ptimization algorithm [35] for training a support vector machine (SMV). The optimized parameters are shown in Table 5. The complexity parameter determines the tradeoff between the model complexity and the degree to which deviations larger than ε (the round-off error that has a fixed value of 1E-12) are tolerated in the optimization procedure. The kernel function is the core of the support vector classifier, allowing it to handle non-linearly separable data sets by adding an additional dimension.

**Table 5:** Optimized parameters for *S*equential *M*inimal *O*ptimization algorithm (SMO)

*Complexity parameter:*	3
*Kernel:*	Radial Basis Function (exp(-*γ|x-y|*^2^)
*γ parameter:*	0.3
*Validation method:*	Leave-one-out cross-validation

Because the number of samples in the input data is limited (four strains at four cultivation conditions, yielding 16 different data objects), leave-one-out (LOO) cross-validation was used for evaluating the predictive power of the model. LOO cross-validation involves the sequential omission of each data object from the training set and using all the remaining ones to train the model. The model is then judged on its ability to correctly classify the omitted object. This is repeated for all the objects in the data set. This method of cross-validation ensures that the maximum amount of data is used for the training of the model, which is particularly important when analyzing a small number of samples, as in our case.

## Authors' contributions

IK carried out the calculations and modelling; ZY performed the fungal fermentations and helped in the metabolome analysis experiments; GPA constructed the recombinant strains and performed the metabolome analysis experiments. SOJ and LO gave valuable suggestions in both the experimental and computational part. IK and GPA participated in the design and coordination of the study, the analysis of results and the writing of the manuscript. All the authors have read and approved the final version of the manuscript.

## Supplementary Material

Additional file 1 The profile of identified intracellular amino and non-amino organic acids, expressed in peak areas normalized to the mass of biomass. Click here for file
