# Supplementary material for: Identification of biomarkers for genotyping Aspergilli using non-linear methods for clustering and classification
Source: BMC Bioinformatics. 2008 Jan 28;9:59. doi: 10.1186/1471-2105-9-59 (PMC2248563; doi:10.1186/1471-2105-9-59)
Supplement: Additional file 1 — The profile of identified intracellular amino and non-amino organic acids, expressed in peak areas normalized to the mass of biomass. [file 1471-2105-9-59-S1.pdf]

	6M (GLU)	6M (XYL)	6M (ETH)	6M (GLR)	6MP (GLU)	6MP (XYL)	6MP (ETH)	6M (GLR)	A4 (GLU)	A4 (XYL)	A4 (ETH)	A4 (GLR)	P (GLU)	P (XYL)	P (ETH)	P (GLR)
M1	0	0	0	0	0	0	0	0	12566	36851	46510	54843	0	0	0	0
M2	2528	2633	2176	1956	1043	3779	4453	3162	12471	31606	36179	46767	0	6634	9429	5959
M3	0	3106	2906	0	0	0	0	0	928	5911	18540	3201	4581	11976	0	6858
M4	0	36017	52201	48959	0	45434	41470	41168	0	0	91697	47873	0	0	29281	96369
M5	0	0	0	0	0	0	0	0	1676	4430	6820	4700	0	0	0	0
M6	48864	13445	0	0	0	0	0	0	0	0	46178	0	13743	22590	33948	0
M7	0	0	0	0	0	0	0	0	0	0	2219	0	0	0	0	0
M8	0	0	0	0	0	0	0	0	0	0	7384	7908	0	0	0	0
M9	0	0	0	0	0	0	0	0	11393	43393	55291	60442	0	0	0	0
M10	0	1027	0	0	0	0	0	0	0	1579	702	795	0	0	0	0
M11	2687242	1992277	437085	1419285	2377118	740171	1683160	1299912	1547593	336114	443715	1028744	1648646	748865	809185	793005
M12	0	0	0	0	0	0	0	0	0	0	23082	0	0	0	0	0
M13	0	0	0	0	0	0	0	0	0	0	50742	0	0	0	0	0
M14	0	0	0	0	0	0	0	0	0	6016	7496	10970	0	0	0	0
M15	2226	3919	2693	1941	0	0	2980	0	7556	21316	57630	56317	1684	3998	5393	2997
M16	0	0	0	0	0	0	0	0	1215	3821	2811	3717	0	0	0	0
M17	63985	49684	15234	36863	61910	0	30430	0	16069	9039	8285	0	18240	10919	19791	20406
M18	0	0	0	0	0	0	0	0	0	0	8522	0	0	0	0	0
M19	489	1486	0	0	0	0	0	0	441	1135	5618	3566	0	0	0	0
M20	388037	375296	290814	401802	357894	196503	319972	428025	135033	88135	146032	174572	353818	147769	280285	185203
M21	0	12600	5119	5705	11895	4748	6433	0	701	0	3436	2620	1723	2251	2326	2174
M22	0	0	0	0	0	0	0	0	0	0	41676	0	25368	0	0	75644
M23	8167	29011	22233	0	2817	0	30174	0	7520	44939	124539	83079	72077	166264	207753	184816
M24	0	0	0	0	0	0	0	0	825	1965	7704	3140	0	0	0	0
M25	0	0	0	0	0	0	0	0	0	44290	44916	0	0	0	0	0
M26	0	0	0	0	0	0	0	0	14693	36337	53303	18973	0	0	0	0
M27	0	0	0	0	0	0	0	0	4621	33682	40816	0	0	0	0	0
M28	145396	196345	453486	222572	185204	124639	154711	229239	55429	0	135272	146208	108270	124942	124294	0
M29	609	0	0	0	0	0	0	0	0	0	435	0	0	0	0	0
M30	0	0	0	0	0	0	0	0	0	0	56059	0	0	0	0	0
M31	12448	24617	21938	12224	2838	7707	24025	13815	5622	56841	234490	62585	98719	215678	245336	160352
M32	575702	473411	61698	459339	483884	179992	105244	466843	84994	64125	84670	98352	269834	137306	114068	192685
M33	0	0	0	0	0	0	0	0	0	0	1740	0	0	0	0	0
M34	13754	53798	0	0	0	0	0	0	24518	47746	160835	0	0	0	0	0
M35	275145	0	78866	84309	163137	94173	150678	72071	79896	29196	69503	25581	113915	60509	55699	40682
M36	31345	28241	17820	0	7164	6706	0	0	7690	11717	47195	10389	4883	9799	0	0
M37	0	0	0	0	0	0	0	0	0	0	722	0	151	0	0	0
M38	1758	13540	0	18357	14977	0	0	22529	4745	6235	16897	3953	7935	9678	5282	0
M39	48133	63136	29671	66209	0	0	0	39794	24682	21225	4609	0	44036	40684	21711	65788
M40	0	0	0	0	0	0	0	0	5650	8951	14725	0	0	0	0	0
M41	0	0	0	0	0	0	0	0	0	0	47317	0	0	0	0	0

	6M (GLU)	6M (XYL)	6M (ETH)	6M (GLR)	6MP (GLU)	6MP (XYL)	6MP (ETH)	6M (GLR)	A4 (GLU)	A4 (XYL)	A4 (ETH)	A4 (GLR)	P (GLU)	P (XYL)	P (ETH)	P (GLR)
M42	0	0	0	0	0	0	0	0	17466	63601	122533	69934	0	0	0	0
M43	0	0	0	0	0	0	0	0	0	0	388172	0	0	0	0	0
M44	1053513	4356438	936486	2921748	3069499	2015892	2053829	2812874	74741	1002463	887635	635470	96033	620026	363086	1525778
M45	1195917	1486629	336878	916813	942986	596180	0	1042435	316350	705531	992035	463549	517551	820240	488677	0
M46	1573645	1232170	82	816723	1255884	169483	111913	169553	283327	462851	390963	721830	474802	508538	440585	673436
M47	0	82730	58211	143050	68680	145549	100188	74078	55710	111847	132171	30666	7136	11002	40542	100794
M48	4555	7948	0	3941	0	0	6098	3948	1287	16072	39055	21399	12723	41981	44601	27486
M49	0	0	0	0	0	0	0	0	3137	13489	12261	2969	1434	0	0	0
M50	0	0	0	0	0	0	0	0	0	0	5423	0	0	0	0	0
M51	0	214	731	616	0	683	80	0	0	0	3543	0	0	0	0	0
M52	3707401	4662598	788739	6587404	6111168	2344089	2630604	7719857	489971	845304	803844	1504920	958898	1137280	898405	2956620
M53	0	0	0	0	0	0	0	0	17656	59110	115234	68573	0	0	0	0
M54	0	0	0	0	0	0	0	0	619	0	316	0	1156	0	0	427
M55	965	2428	1674	4031	1421	0	2792	3493	906	1848	1168	494	0	0	549	1711
M56	0	0	0	0	0	0	0	0	0	0	27974	0	0	0	0	0
M57	0	1896	1732	0	0	0	0	0	0	12419	13953	3650	2451	12165	0	17952
M58	9682	11260	0	0	0	0	6968	14801	0	2948	6734	1461	0	3657	0	4328
M59	0	0	0	8968	6830	0	0	9894	0	0	8248	0	0	0	0	0
M60	2904	0	0	0	0	0	0	0	0	3288	2951	0	0	1899	3075	3962
M61	0	0	0	4844	0	0	26497	0	0	0	18291	0	0	0	4571	0
M62	0	0	0	0	0	0	0	0	8041	23398	69700	24745	0	0	0	0
M63	0	0	0	0	0	0	0	0	0	20743	14444	0	0	0	6436	0
M64	0	0	0	0	0	0	0	0	5190	16556	70818	0	0	0	0	0
M65	0	0	0	0	0	0	0	0	0	0	116909	0	0	0	0	0
M66	0	0	8350	5289	6943	0	0	0	0	0	17184	8044	2219	0	0	10660
M67	0	0	10561	0	0	0	0	0	0	0	7827	0	0	0	0	15337
M68	0	0	0	0	0	0	0	0	5806	0	13687	0	0	0	0	0
M69	305997	841925	485938	1139419	716768	872679	1466260	1068598	44996	293297	570213	343485	154386	398018	363243	988969
M70	0	0	0	0	0	0	0	0	34065	365297	484030	0	0	0	0	0
M71	0	0	0	0	0	0	0	0	0	0	4389	0	0	0	1204	0
M72	0	0	0	0	0	0	0	0	0	5516	23801	37348	0	0	0	0
M73	107507	113341	98721	153798	98228	0	0	187007	78484	45119	108019	70035	162679	0	115690	142756
M74	0	0	0	0	0	0	0	0	0	69448	105743	112485	0	84257	0	75349
M75	20609	72770	0	80507	44631	284968	100361	0	23102	0	111469	20269	15678	35125	142631	45157
M76	0	0	0	9084894	0	0	0	8811887	0	738679	1488871	0	0	0	0	0
M77	0	0	0	0	0	0	0	0	0	363886	292600	0	108632	0	0	0
M78	2025	0	6203	6449	2006	0	11588	24280	0	0	2421	1447	1494	6154	1850	10642
M79	0	0	0	0	0	0	0	0	1097	2451	774	0	0	0	242	0
M80	0	0	0	0	0	0	0	0	0	0	2112	0	0	0	0	0
M81	8413	19829	15958	21805	16478	20341	22320	22202	17153	57365	75325	77034	28668	47452	42403	96156
M82	0	0	0	0	0	0	0	0	0	0	2458	0	0	0	0	0

	6M (GLU)	6M (XYL)	6M (ETH)	6M (GLR)	6MP (GLU)	6MP (XYL)	6MP (ETH)	6M (GLR)	A4 (GLU)	A4 (XYL)	A4 (ETH)	A4 (GLR)	P (GLU)	P (XYL)	P (ETH)	P (GLR)
M83	0	0	0	0	0	0	0	0	0	0	1999	0	0	0	0	0
M84	46363	114387	0	144814	86640	142595	143706	161074	2231	5915	7910	5827	22116	43815	41639	69551
M85	0	0	0	0	0	0	0	0	0	0	3370	0	0	0	0	0
M86	0	269726	1653898	966341	0	0	642365	1565636	0	9621	19703	62660	11543	27912	0	0
M87	0	0	0	0	0	0	0	0	0	0	3590	3697	0	0	0	0
M88	0	0	0	0	0	0	0	0	0	0	22603	0	0	0	0	0
M89	113646	73674	58415	81797	120975	32751	141426	0	59207	20724	56841	30317	100133	46218	71835	45672
M90	0	0	0	0	0	0	0	0	0	0	6747	0	0	0	0	0
M91	0	0	0	0	0	0	0	0	4669	11954	11727	9733	0	0	0	0
M92	0	991	1205	0	0	0	0	0	604	2081	1259	5064	264	0	0	0
M93	0	0	0	0	0	0	0	0	0	0	1854	0	0	0	0	0
M94	0	0	0	0	0	0	0	0	0	0	34246	0	0	0	0	0
M95	0	0	0	0	0	0	0	0	0	0	2731	0	0	0	0	0
M96	0	0	0	0	0	0	0	0	0	0	2260	0	0	0	0	0
M97	0	0	0	0	0	0	0	0	0	391207	575108	0	0	0	0	0
M98	0	8336	0	7232	20955	0	0	0	0	3582	2244	0	0	5868	0	0
M99	0	0	0	0	0	0	0	0	10324	40383	76894	0	0	0	0	0
M100	0	0	0	0	0	0	0	0	0	0	3323	0	0	0	0	0
M101	0	0	0	0	0	0	0	0	0	0	8105	0	0	0	0	0
M102	0	0	0	0	0	0	0	7513	0	529	520	1242	32	894	167	183
M103	0	5432	0	3443	0	0	0	0	0	0	6287	0	0	0	1722	4209
M104	0	0	0	0	0	0	0	0	1861	0	0	9147	0	0	0	0
M105	0	0	0	0	0	0	0	0	0	0	0	1883	0	0	0	0
M106	0	0	0	0	0	0	0	0	0	4460	0	2379	0	0	0	0
M107	0	0	0	0	0	0	0	0	0	2401	0	2169	0	0	0	0
M108	0	0	0	0	0	0	0	0	0	0	0	30130	0	0	0	0
M109	0	0	0	0	0	0	0	0	0	0	0	2734	0	0	0	0
M110	4947	0	0	10963	0	0	0	4579	0	0	0	95137	12510	0	7751	64556
M111	0	0	0	0	0	0	0	0	8289	0	0	36931	0	0	0	0
M112	0	0	0	0	0	1741	0	0	0	0	0	38641	1238	4042	0	0
M113	0	0	0	0	0	0	0	0	0	0	0	62701	0	0	0	0
M114	0	0	0	0	0	0	0	0	0	0	0	56682	0	0	0	0
M115	0	10183	21811	16364	0	7126	5899	7512	7938	0	0	7117	12133	4144	0	0
M116	0	0	0	0	0	0	0	0	0	0	0	11589	0	0	0	0
M117	0	0	0	0	0	0	0	0	0	0	0	24786	0	0	0	0
M118	53472	0	0	10970	22773	0	0	0	10870	0	0	9662	34687	8823	0	6188
M119	0	0	0	0	0	0	0	0	0	0	0	16639	24371	0	0	0
M120	0	0	0	0	0	0	0	0	0	0	0	31913	0	0	0	0
M121	0	0	0	0	0	0	0	0	0	0	0	9985	0	0	0	0
M122	0	0	0	0	0	0	0	0	0	0	0	12093	0	0	0	5624
M123	0	0	0	0	0	0	0	0	0	0	0	4698	0	0	0	0

	6M (GLU)	***6M (XYL)***	***6M (ETH)***	***6M (GLR)***	***6MP (GLU)***	***6MP (XYL)***	***6MP (ETH)***	***6M (GLR)***	***A4 (GLU)***	***A4 (XYL)***	***A4 (ETH)***	***A4 (GLR)***	***P (GLU)***	***P (XYL)***	***P (ETH)***	***P (GLR)***
M124	0	0	0	0	0	0	0	0	0	0	0	2452	0	1746	0	0
M125	0	0	0	0	0	0	0	0	0	0	0	3248	0	0	0	0
M126	0	0	0	0	0	0	0	0	0	0	0	20690	0	11220	0	41264
M127	0	0	0	0	0	0	0	0	0	2362	0	3777	747	0	0	0
M128	0	0	0	0	0	0	0	0	0	0	0	5076	0	0	0	0
M129	0	0	0	0	0	0	0	0	0	0	0	9994	0	0	0	0
M130	0	0	0	0	0	0	0	0	0	0	0	1963	0	1639	0	0
M131	0	0	0	0	0	0	0	0	15707	12303	0	18236	0	10941	22492	22130
M132	0	48564	6634	31409	0	15202	7270	12571	34156	26421	0	15869	50374	29417	43626	12084
M133	23981	0	131674	0	0	0	0	0	8779	12657	0	17809	31584	13991	17582	25386
M134	0	0	0	0	0	0	0	0	0	0	0	3595	0	0	0	0
M135	0	0	2890	0	0	0	0	0	0	0	0	3855	0	0	0	0
M136	0	0	0	0	0	0	0	0	0	0	0	2206	0	0	0	0
M137	37439	0	0	0	0	0	0	0	84117	0	0	40138	40504	0	97825	10566
M138	0	0	0	0	0	0	0	0	0	0	0	71020	0	0	0	0
M139	0	0	0	0	0	0	0	0	0	1948	0	1248	0	0	0	0
M140	32624	54702	201495	86353	52218	0	44704	97187	20879	21261	0	34207	38329	39671	38965	98047
M141	0	0	0	0	0	3437	0	0	0	0	0	865	0	0	946	0
M142	0	0	0	0	0	0	0	0	339011	874239	0	117671	0	0	0	0
M143	0	0	0	0	0	0	0	0	4210	0	0	12000	0	0	0	0
M144	0	0	0	0	0	0	0	0	0	0	0	1959	0	0	0	0
M145	795	0	0	0	0	1080	0	0	0	434	0	448	0	0	0	0
M146	0	0	0	0	0	0	0	0	0	0	0	10854	0	0	0	0
M147	0	0	0	0	0	0	0	0	0	0	0	21226	4201	0	0	23891
M148	0	0	0	0	0	0	0	0	0	0	0	6164	0	0	0	0
M149	0	0	0	0	0	0	0	0	0	0	0	12350	0	0	0	0
M150	0	0	0	0	0	0	0	0	0	0	0	13495	0	0	0	25777
M151	0	0	0	0	0	0	0	0	0	0	0	6862	0	0	0	0
M152	0	0	0	0	0	0	0	0	0	0	0	66343	0	0	0	0
M153	0	0	0	0	0	0	0	0	0	0	0	9945	0	0	0	0
M154	0	0	0	0	0	0	0	0	0	0	0	3547	0	0	0	0
M155	0	0	0	0	0	0	0	0	572	1997	0	602	0	0	0	0
M156	0	0	0	0	36726	0	0	0	32083	0	0	0	40276	0	19961	0
M157	1000825	0	0	0	227219	0	0	0	190299	0	0	0	96395	0	77141	0
M158	55793	0	0	0	0	0	0	0	40853	0	0	0	86507	0	0	0
M159	35312	1689	0	0	248	0	0	0	31	0	0	0	0	0	0	0
M160	0	0	0	0	0	167	0	0	1654	0	0	0	0	0	0	0
M161	0	0	0	0	0	0	0	0	2148	0	0	0	0	0	0	0
M162	0	0	0	0	0	0	0	0	5909	0	0	0	0	0	0	0
M163	1121481	170886	0	102015	264269	0	159672	0	260511	0	0	0	521301	165789	146408	65263
M164	0	0	0	0	0	0	0	0	2082	0	0	0	0	0	0	0

	6M (GLU)	*6M (XYL)*	*6M (ETH)*	*6M (GLR)*	*6MP (GLU)*	*6MP (XYL)*	*6MP (ETH)*	*6M (GLR)*	*A4 (GLU)*	*A4 (XYL)*	*A4 (ETH)*	*A4 (GLR)*	*P (GLU)*	*P (XYL)*	*P (ETH)*	*P (GLR)*
M165	0	0	0	0	0	0	0	0	80169	0	0	0	0	0	0	0
M166	121465	0	0	0	127899	0	0	0	38109	0	0	0	110683	41303	0	0
M167	0	0	0	0	0	0	0	0	506	0	0	0	0	0	0	0
M168	0	0	0	0	0	0	0	0	2363	0	0	0	2694	0	0	0
M169	147159	0	0	117238	67079	0	0	0	14746	0	0	0	30344	0	0	0
M170	0	0	0	0	0	0	0	0	5652	0	0	0	0	0	0	0
M171	0	0	0	0	0	0	0	0	39974	0	0	0	113036	0	0	0
M172	0	0	0	0	0	0	0	0	1779	0	0	0	0	0	0	0
M173	0	0	0	0	0	0	0	0	656	0	0	0	0	0	0	0
M174	0	0	0	0	0	0	0	0	1210	0	0	0	0	0	0	0
M175	0	0	0	0	0	0	0	0	6887	0	0	0	0	0	0	0
M177	0	0	0	0	0	0	0	0	699	1215	0	0	0	0	0	0
M178	0	0	0	0	0	0	0	0	9801	0	0	0	0	0	0	0
M179	0	0	0	0	0	0	0	0	1050	0	0	0	0	0	0	0
M180	0	0	2046	0	0	0	0	0	3585	0	0	0	6402	0	0	0
M181	0	0	0	0	0	0	0	0	1163	0	0	0	0	0	0	0
M182	19059	0	0	0	0	0	0	0	6945	0	0	0	8724	0	0	0
M183	77485	0	0	0	0	0	0	0	31721	0	0	0	14664	0	0	0
M184	0	0	0	0	0	0	0	0	1770	0	0	0	4118	0	0	0
M185	0	0	0	0	0	0	0	0	613	0	0	0	0	0	0	0
M186	0	0	0	0	0	0	0	0	320	0	0	0	1698	0	0	0
M187	0	0	0	0	0	0	0	0	16165	40718	0	0	0	0	0	0
M188	0	0	0	0	0	0	0	0	1203	1799	0	0	0	0	0	0
M189	61580	58598	127194	201629	90274	0	80240	351039	39146	0	0	0	92630	22389	49124	173942
M190	0	0	0	0	0	0	0	0	24022	0	0	0	0	0	0	0
M191	0	33696	0	0	0	0	42664	0	10665	0	0	0	19936	0	0	0
M192	0	0	0	0	342	0	1446	0	1161	1610	0	0	0	0	327	0
M193	10806	0	0	0	0	0	0	0	6498	0	0	0	8290	0	0	0
M194	0	0	1809	2221	0	0	0	0	1439	2755	0	0	0	0	0	992
M195	0	0	0	0	0	0	0	0	540	1534	0	0	0	0	0	0
M196	0	0	0	0	0	0	0	0	7957	0	0	0	0	0	0	0
M197	0	0	0	0	0	0	0	0	499	0	0	0	0	0	0	0
M198	0	0	0	0	0	0	0	0	3247	0	0	0	4312	0	1741	0
M199	0	0	0	0	0	0	0	0	2156	0	0	0	0	0	0	0
M200	0	0	0	0	0	0	0	0	850	0	0	0	0	0	0	0
M201	0	0	0	0	0	0	0	0	1154	0	0	0	0	0	0	0
M202	0	0	0	0	0	0	0	0	3168	0	0	0	2348	0	0	0
M203	0	0	0	0	0	0	0	0	3709	0	0	0	0	0	0	0
M204	290882	150007	0	0	0	0	0	0	43429	0	0	0	0	0	0	0
M205	0	0	0	0	0	0	120	63	99	0	0	0	1090	0	0	0
M206	0	0	0	0	0	0	0	0	0	38400	0	0	0	0	0	0

	6M (GLU)	***6M (XYL)***	***6M (ETH)***	***6M (GLR)***	***6MP (GLU)***	***6MP (XYL)***	***6MP (ETH)***	***6M (GLR)***	***A4 (GLU)***	***A4 (XYL)***	***A4 (ETH)***	***A4 (GLR)***	***P (GLU)***	***P (XYL)***	***P (ETH)***	***P (GLR)***
M207	0	1076	0	0	0	0	0	0	0	1756	0	0	0	0	0	0
M208	0	0	0	0	0	0	0	0	0	49758	0	0	0	0	0	0
M209	0	0	0	0	0	0	0	0	0	22302	0	0	0	0	0	0
M210	0	0	0	0	0	0	0	0	0	5147	0	0	0	0	0	0
M211	0	0	0	0	0	0	0	0	0	10788	0	0	0	0	0	0
M213	0	0	0	0	0	0	0	0	0	5757	0	0	0	17532	0	0
M214	0	0	0	0	0	0	0	0	0	4837	0	0	0	0	0	0
M215	0	0	0	0	0	0	0	0	0	18225	0	0	0	0	7147	0
M216	0	0	0	0	0	0	0	0	0	267816	0	0	0	0	0	0
M217	0	0	0	0	0	0	0	0	0	10681	0	0	0	0	0	0
M218	0	0	0	0	0	0	0	0	0	5402	0	0	0	0	0	0
M219	0	0	0	0	0	0	0	0	0	5185	0	0	0	0	0	0
M220	0	0	0	0	0	0	0	0	0	899	0	0	0	0	0	0
M221	0	0	0	0	0	0	0	0	0	3296	0	0	0	0	0	0
M222	0	0	0	0	0	0	0	0	0	3786	0	0	0	0	0	0
M223	0	0	0	0	0	0	0	0	0	14528	0	0	0	0	0	0
M224	0	0	0	0	0	0	0	0	0	2347	0	0	0	0	0	0
M225	0	0	0	0	0	0	0	0	0	16590	0	0	0	0	0	0
M226	0	0	0	0	0	0	0	0	0	27346	0	0	0	0	0	0
M227	0	0	0	0	0	0	0	0	0	980	0	0	0	0	0	0
M228	0	0	0	0	0	0	0	0	0	1921	0	0	0	0	0	0
M229	0	671	0	0	1472	1088	0	0	0	166	0	0	129	142	0	177
M230	0	0	0	0	0	0	0	0	0	6257	0	0	0	0	0	0
M231	0	0	0	0	0	0	0	0	0	2469	0	0	0	0	0	0
M232	0	0	0	0	0	0	0	0	0	20553	0	0	0	0	0	0
M233	0	8770	0	0	3948	4520	8673	0	0	4929	0	0	0	0	0	0
M234	3930	0	14571	13520	7685	0	14373	0	0	0	0	0	1689	0	2831	8688
M235	0	0	0	0	0	0	0	0	0	0	0	0	8001	0	8038	0
M236	0	0	0	0	0	0	0	0	0	0	0	0	0	0	1986	0
M237	0	0	0	0	0	0	0	0	0	0	0	0	0	0	1499	0
M238	0	0	0	0	0	0	0	0	0	0	0	0	5053	5408	6532	10358
M239	0	0	0	0	0	0	0	0	0	0	0	0	0	0	53307	0
M240	0	0	0	0	0	0	0	0	0	0	0	0	0	0	5205	0
M241	0	0	0	0	0	0	0	0	0	0	0	0	0	0	13258	0
M242	0	0	0	0	0	0	0	0	0	0	0	0	0	0	39478	159
M243	0	0	0	0	0	0	0	0	0	0	0	0	0	0	120805	0
M244	0	0	0	0	0	0	0	0	0	0	0	0	0	0	41295	0
M245	0	0	0	0	0	0	0	0	0	0	0	0	717	0	609	0
M246	0	0	0	0	0	0	0	0	0	0	0	0	0	0	7189	0
M247	0	0	0	0	0	0	0	0	0	0	0	0	0	0	158596	0
M248	26465	0	0	0	0	0	0	0	0	0	0	0	0	0	17315	0

	6M (GLU)	6M (XYL)	6M (ETH)	6M (GLR)	6MP (GLU)	6MP (XYL)	6MP (ETH)	6M (GLR)	A4 (GLU)	A4 (XYL)	A4 (ETH)	A4 (GLR)	P (GLU)	P (XYL)	P (ETH)	P (GLR)
M249	0	0	0	0	0	0	0	0	0	0	0	0	1150	0	1211	0
M250	39783	0	0	39035	52140	0	0	37091	0	0	0	0	12510	0	13158	0
M251	0	0	0	0	0	0	0	0	0	0	0	0	2867	0	2702	0
M252	0	0	0	0	0	0	0	0	0	0	0	0	0	485	1386	2024
M253	0	0	0	0	0	0	0	0	0	0	0	0	0	0	11591	0
M254	22950	27794	0	33422	0	0	0	35378	0	0	0	0	42993	62674	17519	0
M255	0	0	0	23182	0	0	0	26896	0	0	0	0	0	10289	8506	18191
M256	0	0	0	0	0	0	0	0	0	0	0	0	0	0	642	2331
M257	0	29101	18245	0	37408	192	51277	0	0	0	0	0	0	0	36	0
M258	0	0	0	0	0	0	0	0	0	0	0	0	0	1253	2272	0
M259	0	0	0	0	0	0	0	0	0	0	0	0	0	5579	6876	0
M260	0	0	0	0	0	0	0	0	0	0	0	0	20515	0	19129	0
M261	0	0	0	0	0	3012	0	0	0	0	0	0	0	1387	2170	0
M262	0	0	0	0	0	0	0	0	0	0	0	0	0	0	1332	0
M263	0	0	0	0	0	0	0	0	0	0	0	0	0	0	2592	0
M264	0	53113	0	0	0	0	0	0	0	0	0	0	0	0	0	13783
M265	0	0	0	0	0	0	0	0	0	0	0	0	0	0	0	2854
M266	0	0	0	0	0	0	0	0	0	0	0	0	0	0	0	9199
M267	0	0	0	0	0	0	0	0	0	0	0	0	0	0	0	57647
M268	0	0	0	0	0	0	0	0	0	0	0	0	0	0	0	4272
M269	0	0	0	0	0	0	0	0	0	0	0	0	0	61781	0	80975
M270	0	0	0	0	0	0	0	0	0	0	0	0	0	0	0	36552
M271	0	0	0	0	0	0	0	0	0	0	0	0	0	0	0	3503
M272	0	0	0	0	0	0	0	0	0	0	0	0	0	0	0	50158
M273	0	0	0	0	0	0	0	0	0	0	0	0	0	0	0	13353
M274	0	0	0	0	0	0	0	0	0	0	0	0	0	0	0	2024
M275	0	0	0	0	0	0	0	0	0	0	0	0	0	0	0	2576
M276	0	0	0	0	0	0	0	0	0	0	0	0	487	0	0	1625
M277	0	0	0	70608	0	0	0	0	0	0	0	0	24211	0	0	99128
M278	0	0	0	0	0	0	0	0	0	0	0	0	0	0	0	9237
M279	0	0	0	0	0	0	0	0	0	0	0	0	1821	0	0	6197
M280	151	0	0	0	0	0	0	0	0	0	0	0	0	1664	0	1218
M281	0	0	101560	0	437970	0	47681	0	0	0	0	0	0	0	0	59827
M282	46924	0	95623	0	0	0	0	0	0	0	0	0	0	0	0	62131
M283	0	0	0	0	0	0	0	0	0	0	0	0	0	0	0	32662
M284	0	0	0	0	0	9843	0	0	0	0	0	0	0	0	0	35309
M285	0	0	0	0	0	0	0	0	0	0	0	0	0	0	0	7665
M286	0	0	0	0	0	0	0	0	0	0	0	0	0	0	0	13234
M287	6065	82747	60606	64254	0	0	47335	55436	0	0	0	0	0	0	0	79029
M288	0	0	0	0	0	0	0	0	0	0	0	0	0	0	0	2757
M289	0	0	0	0	0	0	0	0	0	0	0	0	0	0	0	2320

	6M (GLU)	6M (XYL)	6M (ETH)	6M (GLR)	6MP (GLU)	6MP (XYL)	6MP (ETH)	6M (GLR)	A4 (GLU)	A4 (XYL)	A4 (ETH)	A4 (GLR)	P (GLU)	P (XYL)	P (ETH)	P (GLR)
M290	0	0	0	0	0	0	0	0	0	0	0	0	0	0	0	155168
M291	0	0	0	0	0	0	0	0	0	0	0	0	1602	0	0	9222
M292	0	0	0	0	0	0	0	0	0	0	0	0	0	0	0	24558
M293	0	0	0	0	0	0	0	0	0	0	0	0	0	0	0	485196
M294	0	0	0	0	0	0	0	0	0	0	0	0	0	0	0	5694
M295	9919	0	10208	0	0	0	0	13542	0	0	0	0	0	0	0	14857
M296	0	0	0	0	0	0	0	0	0	0	0	0	0	0	0	2207
M297	0	0	0	0	0	0	0	0	0	0	0	0	0	0	0	1898
M298	0	0	0	0	0	0	0	0	0	0	0	0	0	0	0	7042
M299	0	0	0	0	0	0	0	0	0	0	0	0	6599	0	0	0
M300	0	0	0	0	0	0	0	0	0	0	0	0	42556	0	0	0
M301	0	0	0	0	0	0	0	0	0	0	0	0	2253	0	0	0
M302	0	0	0	0	0	0	0	0	0	0	0	0	9874	0	0	0
M303	0	0	0	0	0	0	0	0	0	0	0	0	4412	0	0	0
M304	0	0	0	0	0	0	0	0	0	0	0	0	112592	0	0	0
M305	0	0	0	0	0	0	0	0	0	0	0	0	41967	0	0	0
M306	0	0	0	0	0	0	0	0	0	0	0	0	58215	0	0	0
M307	0	0	0	0	0	0	0	0	0	0	0	0	1503	0	0	0
M308	0	0	0	0	0	0	0	0	0	0	0	0	25617	0	0	0
M309	0	0	0	0	0	0	0	0	0	0	0	0	14869	10678	0	0
M310	0	0	0	0	0	0	0	0	0	0	0	0	4202	0	0	0
M311	0	0	0	0	0	0	0	0	0	0	0	0	36853	0	0	0
M312	0	0	0	0	0	0	0	0	0	0	0	0	769	0	0	0
M313	0	0	0	0	0	0	0	0	0	0	0	0	17782	0	0	0
M314	0	0	0	386535	391033	0	0	0	0	0	0	0	77277	0	0	0
M315	0	0	0	0	0	0	0	0	0	0	0	0	7666	0	0	0
M316	0	0	0	0	0	0	0	0	0	0	0	0	2746	0	0	0
M317	0	0	0	0	0	0	0	0	0	0	0	0	24240	0	0	0
M318	0	0	0	0	0	0	0	0	0	0	0	0	7459	0	0	0
M319	0	0	2547	0	0	187	0	0	0	0	0	0	38	0	0	0
M320	0	0	0	0	0	0	0	0	0	0	0	0	1356	0	0	0
M321	0	548	0	2851	0	0	0	0	0	0	0	0	335	0	0	0
M322	0	0	0	0	0	0	0	0	0	0	0	0	704	0	0	0
M323	0	0	0	0	0	0	0	0	0	0	0	0	38608	0	0	0
M324	0	0	0	0	0	0	0	0	0	0	0	0	1317	0	0	0
M325	0	0	0	0	0	0	0	0	0	0	0	0	8966	0	0	0
M326	0	0	0	0	0	0	0	0	0	0	0	0	1044	0	0	0
M327	0	0	0	0	0	0	0	0	0	0	0	0	44466	0	0	0
M328	0	0	0	0	0	0	0	0	0	0	0	0	1695	0	0	0
M329	0	0	0	0	0	0	0	0	0	0	0	0	1475	0	0	0
M330	0	0	0	0	0	0	0	0	0	0	0	0	2687	0	0	0

	6M (GLU)	6M (XYL)	6M (ETH)	6M (GLR)	6MP (GLU)	6MP (XYL)	6MP (ETH)	6M (GLR)	A4 (GLU)	A4 (XYL)	A4 (ETH)	A4 (GLR)	P (GLU)	P (XYL)	P (ETH)	P (GLR)
M331	0	0	0	0	0	0	0	0	0	0	0	0	570	0	0	0
M332	0	0	0	0	0	0	0	0	0	0	0	0	0	143631	0	0
M333	0	0	0	0	0	0	0	0	0	0	0	0	0	14238	0	0
M334	0	0	0	0	0	0	0	0	0	0	0	0	0	1852	0	0
M335	0	0	0	0	0	0	0	0	0	0	0	0	0	6605	0	0
M336	0	0	0	21415	0	0	0	0	0	0	0	0	0	18896	0	0
M337	0	0	0	0	0	0	0	0	0	0	0	0	0	3529	0	0
M338	0	0	0	0	0	0	0	0	0	0	0	0	0	4104	0	0
M339	0	0	0	0	0	0	0	0	0	0	0	0	0	69529	0	0
M340	0	0	0	0	0	0	0	0	0	0	0	0	0	672	0	0
M341	0	51238	0	0	0	0	0	0	0	0	0	0	0	30818	0	0
M342	0	0	0	0	0	0	0	0	0	0	0	0	0	2210	0	0
M343	0	0	0	0	0	0	0	0	0	0	0	0	0	1144	0	0
M344	0	6530	0	0	0	0	0	0	0	0	0	0	0	1258	0	0
M345	0	0	0	0	0	0	0	0	0	0	0	0	0	4907	0	0
M346	0	0	6264	0	0	0	0	0	0	0	0	0	0	0	0	0
M347	0	0	24419	0	0	0	0	0	0	0	0	0	0	0	0	0
M348	0	0	1654	5535	0	0	7730	0	0	0	0	0	0	0	0	0
M349	0	0	12942	0	0	0	0	0	0	0	0	0	0	0	0	0
M350	4566606	3777153	3047536	648133	2599212	1356099	2089850	0	0	0	0	0	0	0	0	0
M351	0	0	1651	0	0	0	0	0	0	0	0	0	0	0	0	0
M352	0	0	49185	0	0	0	0	0	0	0	0	0	0	0	0	0
M353	57214	31672	42021	0	22894	0	10674	0	0	0	0	0	0	0	0	0
M354	0	0	4961	0	0	0	0	0	0	0	0	0	0	0	0	0
M355	0	0	21240	0	0	0	0	0	0	0	0	0	0	0	0	0
M356	0	0	17268	11547	7454	0	10612	0	0	0	0	0	0	0	0	0
M357	0	0	52801	0	0	0	0	0	0	0	0	0	0	0	0	0
M358	4514	0	0	0	0	0	0	0	0	0	0	0	0	0	0	0
M359	16911	0	0	0	0	0	0	0	0	0	0	0	0	0	0	0
M360	39671	0	0	0	16202	0	0	0	0	0	0	0	0	0	0	0
M361	2528	0	0	0	0	0	0	0	0	0	0	0	0	0	0	0
M362	18176	0	0	0	0	0	0	0	0	0	0	0	0	0	0	0
M363	31889	0	0	0	33366	0	0	0	0	0	0	0	0	0	0	0
M364	33254	0	0	0	0	0	0	0	0	0	0	0	0	0	0	0
M365	54322	0	0	0	0	0	0	0	0	0	0	0	0	0	0	0
M366	28903	0	0	0	0	0	0	0	0	0	0	0	0	0	0	0
M367	127919	0	0	0	0	0	0	0	0	0	0	0	0	0	0	0
M368	27950	0	0	0	0	0	0	0	0	0	0	0	0	0	0	0
M369	10458	0	0	0	0	0	0	0	0	0	0	0	0	0	0	0
M370	10687	0	0	0	0	0	0	0	0	0	0	0	0	0	0	0
M371	170565	0	0	0	0	0	0	0	0	0	0	0	0	0	0	0

	6M (GLU)	6M (XYL)	6M (ETH)	6M (GLR)	6MP (GLU)	6MP (XYL)	6MP (ETH)	6M (GLR)	A4 (GLU)	A4 (XYL)	A4 (ETH)	A4 (GLR)	P (GLU)	P (XYL)	P (ETH)	P (GLR)
M372	59467	19794	0	0	33867	0	0	0	0	0	0	0	0	0	0	0
M373	35880	0	0	0	0	0	0	0	0	0	0	0	0	0	0	0
M374	4566	0	0	0	0	0	0	0	0	0	0	0	0	0	0	0
M375	641	0	0	0	0	0	0	0	0	0	0	0	0	0	0	0
M376	18060	0	0	0	0	0	0	0	0	0	0	0	0	0	0	0
M377	962	0	0	0	0	0	0	0	0	0	0	0	0	0	0	0
M378	28038	0	0	0	0	0	0	0	0	0	0	0	0	0	0	0
M379	2264	0	0	0	0	0	0	0	0	0	0	0	0	0	0	0
M380	8254	0	0	0	0	0	0	0	0	0	0	0	0	0	0	0
M381	2092	0	0	0	0	0	0	0	0	0	0	0	0	0	0	0
M382	6731	0	0	0	0	0	0	0	0	0	0	0	0	0	0	0
M383	1663	0	0	0	0	0	0	0	0	0	0	0	0	0	0	0
M384	2179	0	0	0	0	0	0	0	0	0	0	0	0	0	0	0
M385	3278	0	0	0	0	0	0	0	0	0	0	0	0	0	0	0
M386	12606	0	0	0	0	0	0	0	0	0	0	0	0	0	0	0
M387	38175	0	0	0	0	0	0	0	0	0	0	0	0	0	0	0
M388	7011	0	0	0	0	0	0	0	0	0	0	0	0	0	0	0
M389	815	0	0	0	0	0	0	0	0	0	0	0	0	0	0	0
M390	35487	0	0	0	0	0	0	0	0	0	0	0	0	0	0	0
M391	96731	0	0	0	0	0	0	0	0	0	0	0	0	0	0	0
M392	673	0	0	0	0	0	0	0	0	0	0	0	0	0	0	0
M393	4330	0	0	0	0	0	0	0	0	0	0	0	0	0	0	0
M394	1284	0	0	0	0	0	0	0	0	0	0	0	0	0	0	0
M395	7087	0	0	0	0	0	0	0	0	0	0	0	0	0	0	0
M396	649	0	0	1140	0	0	0	0	0	0	0	0	0	0	0	0
M397	1153	0	0	0	0	0	0	0	0	0	0	0	0	0	0	0
M398	5631	0	0	0	0	0	0	0	0	0	0	0	0	0	0	0
M399	894	0	0	0	0	0	0	0	0	0	0	0	0	0	0	0
M400	22692	0	0	0	0	0	0	0	0	0	0	0	0	0	0	0
M401	2525	0	0	0	0	0	0	0	0	0	0	0	0	0	0	0
M402	4170	0	0	0	0	0	0	0	0	0	0	0	0	0	0	0
M403	2962	0	0	0	0	0	0	0	0	0	0	0	0	0	0	0
M404	1273	0	0	0	0	0	0	0	0	0	0	0	0	0	0	0
M405	0	2270	0	1530	0	0	0	0	0	0	0	0	0	0	0	0
M406	0	0	0	40661	0	0	0	0	0	0	0	0	0	0	0	0
M407	0	0	0	28254	0	0	0	41764	0	0	0	0	0	0	0	0
M408	0	0	0	4325	0	0	0	0	0	0	0	0	0	0	0	0
M409	0	0	0	26444	0	0	0	0	0	0	0	0	0	0	0	0
M410	0	0	0	6125	0	0	0	5306	0	0	0	0	0	0	0	0
M411	0	0	0	38540	0	0	0	0	0	0	0	0	0	0	0	0
M412	0	0	0	158250	158469	0	0	0	0	0	0	0	0	0	0	0

	6M (GLU)	6M (XYL)	6M (ETH)	6M (GLR)	6MP (GLU)	6MP (XYL)	6MP (ETH)	6M (GLR)	A4 (GLU)	A4 (XYL)	A4 (ETH)	A4 (GLR)	P (GLU)	P (XYL)	P (ETH)	P (GLR)
M413	0	0	0	202452	0	0	0	0	0	0	0	0	0	0	0	0
M414	0	0	0	4181	0	0	0	0	0	0	0	0	0	0	0	0
M415	0	1823	0	0	0	0	0	0	0	0	0	0	0	0	0	0
M416	0	14943	0	0	0	0	0	0	0	0	0	0	0	0	0	0
M417	0	60091	0	0	0	0	0	0	0	0	0	0	0	0	0	0
M418	0	68831	0	0	0	0	0	0	0	0	0	0	0	0	0	0
M419	0	236185	0	0	0	0	0	0	0	0	0	0	0	0	0	0
M420	0	177374	0	0	0	0	0	0	0	0	0	0	0	0	0	0
M421	0	1768	0	0	0	0	0	0	0	0	0	0	0	0	0	0
M422	0	237601	0	0	302447	0	0	0	0	0	0	0	0	0	0	0
M423	0	2585	0	0	0	0	0	0	0	0	0	0	0	0	0	0
M424	0	23209	0	0	0	0	0	0	0	0	0	0	0	0	0	0
M425	0	6093	0	0	0	0	0	0	0	0	0	0	0	0	0	0
M426	0	227906	0	0	0	0	0	0	0	0	0	0	0	0	0	0
M427	0	5206	0	0	0	0	0	0	0	0	0	0	0	0	0	0
M428	0	19284	0	0	0	0	0	0	0	0	0	0	0	0	0	0
M429	0	14173	0	0	0	0	0	0	0	0	0	0	0	0	0	0
M430	0	235995	0	0	0	0	0	0	0	0	0	0	0	0	0	0
M431	0	1905	0	0	0	0	0	0	0	0	0	0	0	0	0	0
M432	0	13382	0	0	0	0	0	0	0	0	0	0	0	0	0	0
M433	0	0	0	0	0	0	121380	0	0	0	0	0	0	0	0	0
M434	0	0	0	0	0	0	4828	0	0	0	0	0	0	0	0	0
M435	0	0	0	0	0	0	9122	0	0	0	0	0	0	0	0	0
M436	0	0	0	0	0	0	7665	0	0	0	0	0	0	0	0	0
M437	0	0	0	0	0	0	5048	0	0	0	0	0	0	0	0	0
M438	0	0	0	0	0	0	80418	229833	0	0	0	0	0	0	0	0
M439	0	0	0	0	0	0	13223	0	0	0	0	0	0	0	0	0
M440	0	0	0	0	18975	0	0	0	0	0	0	0	0	0	0	0
M441	0	0	0	0	7186	0	0	0	0	0	0	0	0	0	0	0
M442	0	0	0	0	25351	0	0	0	0	0	0	0	0	0	0	0
M443	0	0	0	0	16615	0	0	0	0	0	0	0	0	0	0	0
M444	0	0	0	0	16099	0	0	0	0	0	0	0	0	0	0	0
M445	0	0	0	0	50466	0	0	0	0	0	0	0	0	0	0	0
M446	0	0	0	0	5109	0	0	0	0	0	0	0	0	0	0	0
M447	0	0	0	0	1480	0	0	0	0	0	0	0	0	0	0	0
M448	0	0	0	0	1955	0	0	0	0	0	0	0	0	0	0	0
M449	0	0	0	0	3395	0	0	0	0	0	0	0	0	0	0	0
M450	0	0	0	0	0	0	0	634587	0	0	0	0	0	0	0	0
M451	0	0	0	0	0	0	0	273483	0	0	0	0	0	0	0	0
M452	0	0	0	0	0	6133	0	3606	0	0	0	0	0	0	0	0
M453	0	0	0	0	0	0	0	2228	0	0	0	0	0	0	0	0

	6M (GLU)	6M (XYL)	6M (ETH)	6M (GLR)	6MP (GLU)	6MP (XYL)	6MP (ETH)	6M (GLR)	A4 (GLU)	A4 (XYL)	A4 (ETH)	A4 (GLR)	P (GLU)	P (XYL)	P (ETH)	P (GLR)
M454	0	0	0	0	0	0	0	579554	0	0	0	0	0	0	0	0
M455	0	0	0	0	0	0	0	14145	0	0	0	0	0	0	0	0
M456	0	0	0	0	0	0	0	195745	0	0	0	0	0	0	0	0
M457	0	0	0	0	0	0	0	3463	0	0	0	0	0	0	0	0
M458	0	0	0	0	0	0	0	508305	0	0	0	0	0	0	0	0
M459	0	0	0	0	0	0	0	2921	0	0	0	0	0	0	0	0
M460	0	0	0	0	0	2236	0	0	0	0	0	0	0	0	0	0
M461	0	0	0	0	0	47490	0	0	0	0	0	0	0	0	0	0
M462	0	0	0	0	0	26752	0	0	0	0	0	0	0	0	0	0
M463	0	0	0	0	0	3858	0	0	0	0	0	0	0	0	0	0
M464	0	0	0	0	0	3940	0	0	0	0	0	0	0	0	0	0
